# H263A and SCAN1/H493R mutant TDP1 block TOP1-induced double-strand break repair during gene transcription in quiescent cells and promote cell death

**DOI:** 10.1038/s41419-025-08085-y

**Published:** 2025-11-28

**Authors:** Diana Rubio-Contreras, Daniel Hidalgo-García, Carmen Angulo-Jiménez, Esperanza Granado-Calle, Margarita Sabio-Bonilla, Jose F. Ruiz, Fernando Gómez-Herreros

**Affiliations:** 1 Instituto de Biomedicina de Sevilla (IBiS), Hospital Virgen del Rocío/CSIC/Universidad de Sevilla, Seville, Spain; 2 Departamento de Genética, Facultad de Biología, Universidad de Sevilla, Seville, Spain; 3 Departamento de Bioquímica Vegetal y Biología Molecular, Facultad de Biología, Universidad de Sevilla, Seville, Spain

**Keywords:** DNA adducts, Mechanisms of disease, Double-strand DNA breaks

## Abstract

DNA single-strand break (SSB) repair defects lead to hereditary neurological syndromes. Spinocerebellar ataxia with axonal neuropathy type 1 (SCAN1), is caused by the homozygous H493R mutation in tyrosyl-DNA phosphodiesterase 1 (TDP1), an enzyme that initiates the repair of DNA topoisomerase 1 (TOP1)-induced SSBs by unlinking the TOP1 peptide from the break. Although TDP1 also initiates the repair of TOP1-induced DNA double-strand breaks (DSBs) associated with transcription, the role of TOP1-induced DSBs in SCAN1 pathology remains unclear. Here, we have addressed the impact of the SCAN1/H493R mutation on the repair of TOP1-induced DSBs. We demonstrate that while TDP1 loss delays the repair of these breaks, SCAN1/H493R completely blocks it in RPE-1 quiescent cells. This blockage is specific to DSBs and is accompanied by a prolonged trapping of mutated TDP1 on DNA, but not of TOP1 cleavage complexes (TOP1cc). Intriguingly, the H263A inactivating mutation of TDP1, which accumulates TOP1cc, also blocks TOP1-induced DSB repair. Importantly, both SCAN1/H493R and H263A mutations exhibit genome instability and cell death. Moreover, we demonstrate that tyrosyl-DNA phosphodiesterase 2 (TDP2) can compensate for TDP1 loss in RPE-1 quiescent cells. Collectively, our data support the potential role of TOP1-induced DSBs as a main contributor to certain hereditary neurological syndromes.

## Introduction

DNA topoisomerases are essential enzymes with crucial functions in DNA metabolism [1]. These enzymes release the torsional stress generated in the DNA by processes such as transcription and replication, facilitating DNA transactions. Type IB topoisomerases, such as human TOP1, relax superhelical stress by introducing DNA single-strand breaks (SSBs), allowing one broken DNA strand to rotate relative to the intact strand. TOP1 is thought to be particularly relevant for maintaining genome stability and during transcription [2]. A key intermediate in TOP1 activity is the cleavage complex, in which the DNA is cleaved and the enzyme is covalently bound to the 3’ end of the DNA via a phosphotyrosine linkage [3]. Normally, TOP1 cleavage complexes (TOP1cc) are transient, since TOP1 reseals the break at the culmination of its catalytic cycle. However, DNA lesions, DNA metabolism or exposure to antitumor agents that act as topoisomerase poisons can stabilize TOP1ccs, prolonging their half-life of this intermediary. These situations can lead to the formation of irreversible TOP1cc, commonly known as ‘abortive’ TOP1ccs, that threaten genome integrity.

To be repaired, abortive TOP1ccs are ubiquitinated and degraded by the proteasome, or can also be processed by other proteases [4]. Following proteolysis, the residual TOP1 peptide remains covalently bound to the 3’ end of the DNA break, exposing a 5’ hydroxyl moiety that triggers break signaling by PARP1 and the recruitment of XRCC1 and associated SSB repair factors. Tyrosyl DNA phosphodiesterase 1 (TDP1), a highly conserved enzyme among eukaryotes, is recruited to the TOP1cc-DNA adduct. The coordinated activity of two highly conserved histidine residues of TDP1 enables a two-step catalytic cycle to release the remaining TOP1 adduct. First, H263 performs a nucleophilic attack on the TOP1 phosphotyrosine-DNA adduct, releasing the TOP1 residual peptide and leading to a phosphohistidine-bound TDP1-DNA intermediate. Next, H493 mediates a general acid/base catalytic reaction to break the TDP1-DNA covalent linkage with the participation of a water molecule [5]. Once TDP1 is released, canonical 3’-hydroxyl/5’-phosphate DNA ends are restored by polynucleotide kinase 3’-phosphatase (PNKP), and repair is completed by ligation of the DNA ends by DNA Ligase III (LIG3) [6].

Defects in several SSB repair factors result in hereditary neurological syndromes. These syndromes are characterized, among other common features, by a marked cerebellar cell loss and ataxia [6]. A homozygous mutation in TDP1 causes the rare neurodegenerative syndrome spinocerebellar ataxia with axonal neuropathy type 1 (SCAN1) [7]. SCAN1 is an autosomal recessive neurodegenerative disease characterized by progressive ataxia, cerebellar atrophy, and distal sensorimotor axonal neuropathy. To date, only thirteen patients from three apparently unrelated consanguineous families have been described [7, 8]. Notably, in all these cases, SCAN1 is caused by a homozygous histidine to arginine mutation on H493, the catalytic histidine residue of TDP1 [7, 9]. H493R greatly reduces (~25-fold) the removal of TOP1 from 3’-termini, and SCAN1 cells accumulate TOP1cc [10]. Combined with a 2.5-fold reduction in TDP1 levels in SCAN1 cells, this results in a 60-fold decrease in the first step of TOP1 removal [11, 12]. Additionally, H493R impairs the resolution of the TDP1-DNA phosphohistidyl linkage, resulting in longer-lived covalent TDP1-DNA intermediates [11, 13–15]. Most cellular studies on TOP1-induced DNA damage focus on either the deletion or depletion of TDP1. Consequently, several molecular aspects of SCAN1 pathology remain unclear, including the relative contribution of the loss of TOP1cc unlinking and TDP1 trapping to the disease. This is a pivotal question since abortive TOP1cc seems to be a critical endogenous pathogenic lesion in SSB repair defects and other related diseases, such as ataxia telangectasia [16].

TOP1-induced SSBs can be converted into DNA double-strand breaks (DSBs) in cycling cells due to collisions with the replication machinery. However, replication-independent DSBs can also arise as a result of the TOP1 activity during transcription [17]. These DSBs can be induced by the TOP1 poison camptothecin (CPT), which stabilizes TOP1ccs and is commonly used in cancer treatment [16, 18]. These replication-independent TOP1-induced DSBs are associated with RNA polymerase II transcription and originate from multiple sources, suggesting that they may have a heterogeneous chemical nature [19–21]. Importantly, TDP1 is a key factor in the repair of TOP1-induced DSBs associated with transcription, which are a significant source of genome instability and cell death in quiescent cells [18, 22, 23]. Notably, a very recent study revealed that the SCAN1 mutation hampers the repair of transcriptional DNA double-strand breaks in G1 phase in U2OS cells [24]. However, the precise mechanism underlying TOP1-induced DSB repair blockage in SCAN1 remains unclear. Additionally, the impact of the SCAN1 mutation, compared to TDP1 loss, on TOP1-induced DSB repair, genome instability and survival in quiescent cells remains unstudied. Clarifying these important questions is essential for understanding the potential contribution of TOP1-induced DSBs to SCAN1 neuropathology.

Here, we have directly addressed the role of the SCAN1-causing mutation H493R, in the repair of replication-independent TOP1-induced DSBs in quiescent cells. We show that H493R mutant TDP1 blocks TOP1-induced DSB repair in quiescent cells, resulting in increased genome instability and reduced cellular survival. Strikingly, the H493R mutant TDP1 only blocks alternative TOP1-induced DSB repair pathways when present in the homozygous state. In addition, we demonstrate that TDP2 can backup TDP1 loss in quiescent RPE-1 cells, but TDP2-proficiency is insufficient to facilitate TOP1-induced DSB repair in H493R cells. Intriguingly, a catalytically dead H263A mutation accumulates abortive TOP1cc and results in a more deleterious effect on genome instability and cell survival than either TDP1 loss or the H493R mutation. Our findings clarify the defect in TOP1-induced DSB repair associated with the SCAN1 mutation, providing novel insights into SCAN1 and other SSB-associated neurodegenerative diseases. These results support the idea that defective TOP1-induced DSB repair may significantly contribute to these pathologies.

## Results

### TDP1^H493R^ and TDP1^H263A^ block TOP1-induced DSB repair in quiescent cells

**Fig. 1:**
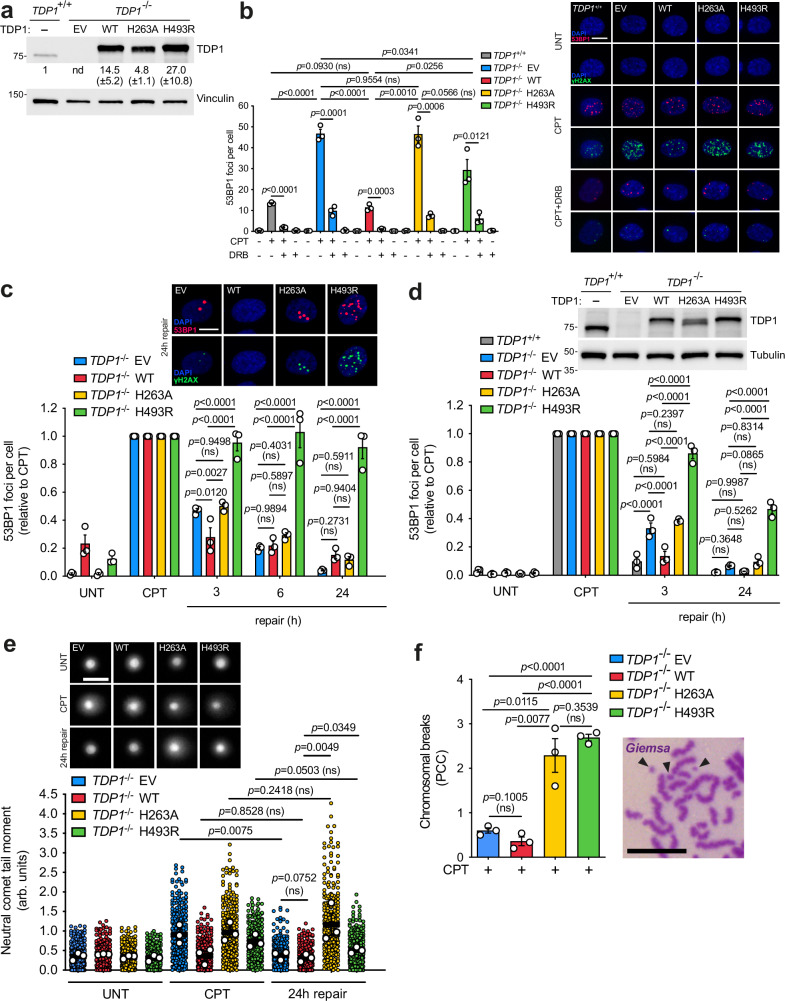
TDP1^H493R^ and TDP1^H263A^ block TOP1-induced DSB repair in RPE-1 quiescent cells. **a** Protein blot of TDP1 in serum-starved *TDP1*^+/+^ and *TDP1*^−/−^ RPE-1 cells complemented with an empty vector (EV), wild-type TDP1 (WT), TDP1^H263A^ (H263A) or TDP1^H493R^ (H493R) is shown. Quantification represented as mean ± SEM (*n* = 3 independent experiments). nd not detectable. **b** 53BP1 foci in serum-starved complemented cells treated with CPT (12.5 μM) for 1 h. Where indicated, cells were pre-incubated with DRB (100 μM) for 3 h prior to CPT treatment. Representative images of 53BP1 (red) and γH2AX (green) foci and DAPI counterstain (blue) are shown. **c**, **d** 53BP1 foci in serum-starved complemented cells after 1 h treatment with 12.5 μM CPT, and during repair in drug-free medium. Representative images for the 24 h of repair time point (**c**) and a protein blot of TDP1 (**d**) are shown. A low doxycycline dose was used in **d**. Other details as in **a** and **b**. **e** Detection of DSBs by neutral comet assay in serum-starved complemented cells treated with CPT (25 μM) for 1 h and after 24 h repair in drug-free medium. Representative images of nuclei are shown. **f** Chromosomal breaks were quantified in serum-starved complemented cells treated with CPT (25 μM) for 2 h followed by 24 h repair in drug-free medium and fused with HeLa cells synchronized in metaphase. A representative image of chromosomal breaks is shown. Black arrows indicate chromosomal breaks. UNT untreated. *n* = 3 independent experiments. Data were represented as mean ± SEM. Statistical significance was determined by two-tailed unpaired *t*-test for **b** and **f**, two-way ANOVA followed by Tukey’s multiple comparisons test for **c** and **d**, and ratio paired *t*-test for **e**. ns non-significance. Scale bar, 10 μm for **b**, **c** and **f** and 50 μm for **e**.

We previously reported that TDP1 is required for the repair of TOP1-induced DSBs in quiescent hTERT-immortalized retinal pigment epithelial cells (RPE-1) cells [23]. To evaluate potential defects in TOP1-induced DSB repair in TDP1-associated neurological disease SCAN1, we conducted complementation experiments in *TDP1*^-/-^ RPE-1 cells using an empty vector (EV), with a wild-type TDP1 (WT), with a histidine-to-alanine mutant in the catalytic histidine 263 of TDP1 (hereafter TDP1^H263A^), reported to be inactive [5, 25], or with the SCAN1-associated mutation H493R (hereafter TDP1^H493R^), which were under the control of a doxycycline-inducible promoter (for details, see methods). To study replication-independent TOP1-associated DSBs, we synchronized cells in G0/G1 by confluency and serum starvation resulting in over 97% of RPE-1 cells arrested in G0/G1 (Supplementary Fig. 1) (for details, see methods) [26]. Twenty-four hours of doxycycline treatment in RPE-1 quiescent cells induced equivalent levels of wild-type TDP1 and TDP1^H493R^, which were overexpressed compared to endogenous TDP1 (Fig. 1a). TDP1^H263A^ expression was significantly lower than that of wild-type TDP1 and TDP1^H493R^, likely due to the previously reported toxicity of TDP1^H263A^ (Fig. 1a) [27–29]. Next, we treated quiescent cells with CPT, which selectively induces abortive TOP1ccs [30]. Notably, exposure to CPT rapidly induced 53BP1 and H2AX serine 139 phosphorylation (hereafter γH2AX) immunofoci, common surrogate markers of DSBs [31] (Fig. 1b). RNA polymerase II elongation inhibition with 5,6-dichloro-1-β-D-ribofuranosylbenzimidazole (DRB) before TOP1 poisoning resulted in ≃80% reduction in 53BP1 foci in all cell lines, in agreement with TOP1-induced DSBs in quiescent RPE-1 cells being dependent on gene transcription [23] (Fig. 1b). Importantly, expression of wild-type TDP1 suppressed the accumulation of CPT-induced DSBs to wild-type levels (*TDP1*^*+/+*^) and to a much greater extent than TDP1^H493R^, which remained close to the levels observed with the EV (Fig. 1b). TDP1^H263A^ expression resulted in similar number of DSBs compared to EV and TDP1^H493R^(Fig. 1b). These results suggest that, similarly to TDP1 deficiency, both TDP1^H263A^ and TDP1^H493R^ lead to the accumulation of transcription-associated CPT-induced DSBs in RPE-1 quiescent cells. Notably, the overexpression of TDP1 mutants did not alter global transcription levels (Supplementary Fig. 2a). Furthermore, we did not observe significant changes in TOP1 levels, which, as previously shown, underwent significant degradation upon CPT treatment [19] (Supplementary Fig. 2b). Degradation was uniform among the mutants, indicating that they did not influence the formation or degradation of abortive TOP1ccs, consistent with the established role of TDP1 (Supplementary Fig. 2b).

Next, we measured TOP1-induced DSB repair rates by following the kinetics of 53BP1 foci after CPT removal in quiescent cells. Wild-type TDP1 expression suppressed the repair defect observed in EV-complemented cells, completing repair three hours after CPT removal (Fig. 1c). In contrast, TDP1^H263A^-expressing cells exhibited a significant repair defect compared to wild-type TDP1, similar to EV-complemented cells, suggesting that TDP1^H263A^ overexpression might slow down TOP1-induced DSB repair. Notably, TDP1^H493R^-expressing cells exhibited a very strong repair defect that extended for six- and twenty-four hours post-treatment, suggesting that, not only TDP1^H493R^ is unable to repair transcription-associated TOP1-induced DSBs in quiescent cells, but it completely blocks the repair process (Fig. 1c). Importantly, we obtained similar results when the doxycycline concentration was reduced, resulting in lower levels of TDP1, TDP1^H263A^ and TDP1^H493R^ expression, comparable to endogenous TDP1 (*TDP1*^*+/+*^), suggesting that the strong repair defect observed in TDP1^H493R^ cells was not due to TDP1^H493R^ overexpression (Fig. 1d).

To directly evaluate TOP1-induced DSB formation and repair, we performed a neutral comet assay, a technique that specifically detects DSBs [32], confirming that TDP1-deficient and TDP1^H493R^-complemented cells accumulate CPT-induced DSBs and that TDP1^H493R^ is unable to resume repair after twenty-four hours (Fig. 1e). Intriguingly, TDP1^H263A^ expression also resulted in a significant TOP1-induced DSBs repair defect, even more severe than in TDP1^H493R^ cells (Fig. 1e). These results were somehow contradictory to repair kinetics and suggest that TDP1^H263A^ blocks TOP1-induced DSBs, although these excess breaks are not detected by 53BP1 foci immunofluorescence or by γH2AX foci which colocalize with 53BP1 (Fig. 1c). To further confirm this observation, we directly visualize DSB in quiescent cells using premature chromosome condensation (see materials and methods and Supplementary Fig. 2c for details). After CPT treatment and repair, quiescent cells were fused to HeLa mitotic cells, promoting the condensation and thus the visualization of single chromatid chromosomes in quiescent cells. TOP1-induced DSB formation was directly measured by visualizing small chromosomal fragments upon CPT treatment and by Giemsa staining, confirming that TDP1^H263A^ cells accumulate CPT-induced DSBs after twenty-four hours of repair (Fig. 1f).

Finally, to examine whether TDP1^H493R^ and TDP1^H263A^ expression also affect the repair rate of SSBs, we quantified nuclear poly(ADP-ribose) (hereafter PAR) levels in cells upon DNA damage by immunofluorescence, which provides an indirect measure of SSB levels [33]. Notably, we detected much higher PAR signal in TDP1^H263A^ and in TDP1^H493R^ than in wild-type TDP1-expressing cells upon CPT treatment (Supplementary Fig. 3). Nevertheless, although the decrease in PAR signal was clearly slower in TDP1^H263A^ and TDP1^H493R^ than in wild-type TDP1-expressing cells, almost all PAR signal had disappeared three hours after repair (Supplementary Fig. 3). These results demonstrate that the blockage of TOP1-induced DSB repair by TDP1^H263A^ and TDP1^H493R^ is much more prolonged in time than the blockage of SSB repair.

### TDP1^H493R^ gets trapped on DNA and blocks TOP1-induced DSB repair

**Fig. 2:**
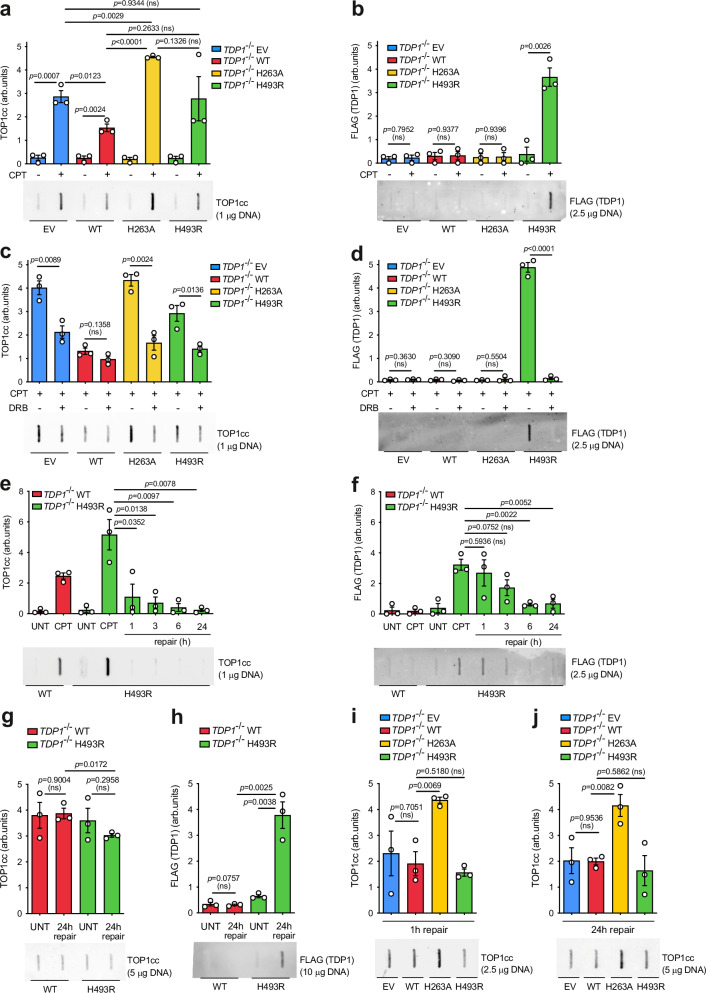
TDP1^H493R^ is covalently trapped on DNA in RPE-1 quiescent cells. Analysis of TOP1 cleavage-complexes (TOP1cc) (**a**, **c**, **e**, **g**, **i**, and **j**) and FLAG (TDP1) (**b**, **d**, **f**, and **h**) by ICE assay. Serum-starved *TDP1*^−/−^ RPE-1 cells complemented with an empty vector (EV), wild-type TDP1 (WT), TDP1^H263A^ (H263A) or TDP1^H493R^ (H493R) were treated with CPT (25 μM) for 1 h followed by repair in drug-free medium where indicated. Where indicated, cells were pre-incubated with DRB (100 μM) for 3 h prior to CPT treatment. *Top*, quantification. *Bottom*, representative plots of TOP1cc and FLAG are shown. UNT untreated. *n* = 3 independent experiments. Data were represented as mean ± SEM. Statistical significance was determined by two-tailed unpaired *t*-test. ns non-significance.

To elucidate the mechanism underlying the blockage of TOP1-induced DSB repair, we measured DNA-protein covalent complexes by in vivo complex of enzyme (ICE) assay [34]. Our cellular model provides a highly useful system for detecting abortive TOP1cc and DNA-TDP1 covalent complexes, as previously shown by Ghosh and collaborators in the study of mitochondrial TOP1 [35]. For the detection of TOP1ccs, we employed an antibody raised against a peptide corresponding to the active site of the TOP1 with a phosphorylated Tyr723 residue [36]. In agreement with previous reports, TDP1-deficient quiescent RPE-1 cells accumulated TOP1ccs upon CPT treatment (Fig. 2a). Notably, the expression of wild-type TDP1, but not of TDP1^H263A^ or TDP1^H493R^, reduced the accumulation of TOP1cc upon CPT treatment in *TDP1*^*−/−*^ cells, suggesting that TDP1^H263A^ and TDP1^H493R^ largely prevent the removal of abortive TOP1ccs (Fig. 2a). We next employed an antibody to detect the complementing FLAG-tagged TDP1 variants. Strikingly, we detected a significant accumulation of covalent DNA-TDP1 complexes in TDP1^H493R^, but not in TDP1 or TDP1^H263A^-expressing cells, demonstrating that H493R mutation covalently traps TDP1 on DNA in quiescent RPE-1 cells upon TOP1 poisoning (Fig. 2b). Importantly, RNA polymerase II elongation inhibition with DRB prior to TOP1 poisoning significantly reduced TOP1cc accumulation (Fig. 2c) and ablated DNA-TDP1^H493R^ covalent trapping (Fig. 2d), in agreement with abortive activity of TOP1 in quiescent RPE-1 cells being dependent on gene transcription.

Next, we measured TOP1cc and DNA-TDP1 covalent complexes during repair in TDP1^H493R^-expressing cells. We observed a fast disappearance of TOP1ccs within the first hour (Fig. 2e). This is consistent with the fact that ICE signal comes from abortive and non-abortive TOP1cc but, considering the difference upon induction observed in wild-type TDP1 overexpressing cells, it also suggests that most of abortive TOP1ccs rapidly diminish following CPT removal (Fig. 2e). Contrary, the levels of DNA-TDP1^H493R^ covalent complexes remained unchanged for one hour and started to decay after three hours (Fig. 2f). These results indicate that DNA-TDP1^H493R^ covalent complexes persist but that, eventually, are either reverted, removed, or degraded. To directly test the persistence of trapped TDP1^H493R^ we increased the DNA load in the assay. Importantly, twenty-four hour after CPT, significant amounts of DNA-TDP1^H493R^ covalent complexes, but not of abortive TOP1cc, remained compared to wild-type TDP1 (Fig. 2g, h). Altogether these results demonstrate that TDP1^H493R^ trapping is not completely irreversible in vivo but, at least in some cases, is persistent.

We next compared post-repair TOP1cc accumulation among all mutants. Notably, after one hour of repair, TOP1ccs were significantly more persistent in TDP1^H263A^ than in EV, wild-type and TDP1^H493R^-expressing cells, indicating that TDP1^H263A^ is unable to efficiently repair abortive TOP1cc (Fig. 2i). More importantly, after twenty-four hours, TDP1^H263A^-expressing cells still retained significant TOP1cc signal compared to wild-type TDP1 (Fig. 2j). These results suggest that, unlike TDP1-deficient and TDP1^H493R^-expressing cells, TDP1^H263A^-expressing cells accumulate abortive TOP1ccs in vivo.

### TDP1^H493R^ and TDP1^H263A^ are recessive mutations

**Fig. 3:**
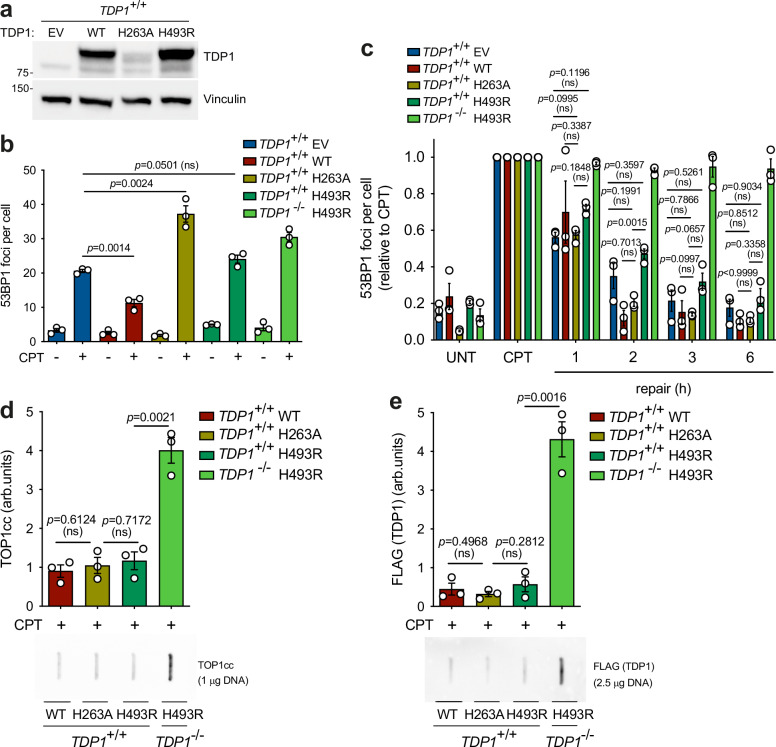
TDP1 suppresses TDP1^H493R^-induced DSB repair blockage. **a** Protein blot of TDP1 in serum-starved *TDP1*^+/+^ RPE-1 cells complemented with an empty vector (EV), wild-type TDP1 (WT), TDP1^H263A^ (H263A) or TDP1^H493R^ (H493R) is shown. **b** 53BP1 foci in serum-starved complemented cells treated with CPT (12.5 μM) for 1 h. **c** 53BP1 foci in serum-starved complemented cells after 1 h treatment with 12.5 μM CPT, and during repair in drug-free medium. **d**, **e** Analysis of TOP1 cleavage-complexes (TOP1cc) (**d**) and FLAG (TDP1) (**e**) by ICE assay. Serum-starved complemented cells were treated with CPT (25 μM) for 1 h. *Top*, quantification. *Bottom*, representative plots of TOP1cc and FLAG are shown. UNT untreated. *n* = 3 independent experiments. Data were represented as mean ± SEM. Statistical significance was determined by two-tailed unpaired *t*-test for (**b**), (**d**) and (**e**), and two-way ANOVA followed by Tukey’s multiple comparisons test for (**c**). ns non-significance.

The blockage of TOP1-induced DSB repair in TDP1^H493R^-expressing cells suggests a potential dominant-negative effect of the H493R mutation. To determine whether this DSB repair blockage is compatible with SCAN1, which is an autosomal recessive syndrome, we overexpressed wild-type TDP1 and TDP1^H493R^ in wild-type RPE-1 cells (hereafter *TDP1*^*+/+*^) (Fig. 3a). The overexpression levels were similar to those observed in *TDP1*^*-/-*^ cells (Supplementary Fig. 4a). We did not observe significant changes in TOP1 levels, which, as previously shown, underwent uniform degradation upon CPT treatment (Supplementary Fig. 4b). Following CPT treatment, wild-type TDP1 overexpression significantly reduced the accumulation of TOP1-induced DSBs, suggesting that endogenous TDP1 might be limiting for the repair of TOP1-induced SSBs and DSBs under the conditions used in this study (Fig. 3b). Notably, TDP1^H493R^-overexpressing cells exhibited very similar levels of DSBs to those of EV-complemented cells when treated with CPT, indicating that TDP1^H493R^ expression in wild-type cells does not lead to a large accumulation of TOP1-induced DSBs (Fig. 3b). More importantly, TDP1^H493R^-overexpressing cells generally exhibited no significant defects in TOP1-induced DSB repair either, demonstrating that TDP1^H493R^ does not block repair when present in heterozygous state (Fig. 3c). Since we had previously associated abortive TOP1cc accumulation and TDP1^H493R^ trapping with TOP1-induced DSB repair blockage, we analyzed TOP1cc and TDP1 covalent complexes by ICE assay. Strikingly, TDP1^H493R^ expression in wild-type cells did not result in either abortive TOP1cc accumulation nor in TDP1^H493R^ trapping (Fig. 3d, e). Given the strong overexpression of TDP1^H493R^, together with our previous observations, these results suggest that the reason of TDP1^H493R^ trapping and DSB blockage are not dominant is dependent on the presence of wild-type TDP1.

To study the dominant or recessive nature of H263A, we also expressed TDP1^H263A^ in wild-type RPE-1 cells (Fig. 3a). TDP1^H263A^ expression was significantly lower than that of wild-type TDP1 and TDP1^H493R^ (Fig. 3a). Notably, TDP1^H263A^-overexpressing cells accumulated more DSBs than EV-complemented cells when treated with CPT, indicating that TDP1^H263A^ expression in heterozygous state can promote the accumulation of TOP1-induced DSBs upon TOP1 poisoning (Fig. 3b). TDP1^H263A^-expressing cells did not show significant repair defects compared to EV, wild-type TDP1 or TDP1^H493R^-overexpressing cells (Fig. 3c). More importantly, TDP1^H263A^ overexpression did not result in a significant increase in abortive TOP1ccs or in DNA-TDP1 complexes (Fig. 3d, e). Despite the observed increase in the accumulation of DSBs upon CPT treatment, these results suggest the recessive nature of TDP1^H263A^.

### TDP2 backs up TDP1 deficiency in quiescent cells

**Fig. 4:**
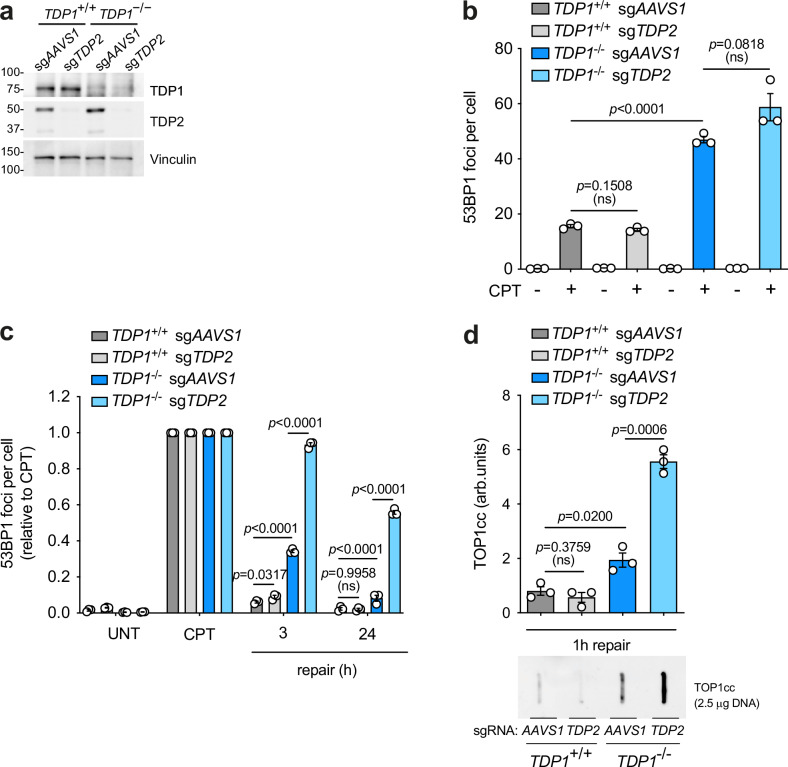
TDP2 backs up TDP1 deficiency. *TDP1*^+/+^ and *TDP1*^−/−^ mock-depleted (sg*AAVS1*) or TDP2-depleted (sg*TDP2*) RPE-1 cells. **a** Protein blot of TDP1 and TDP2 is shown. **b** 53BP1 foci in serum-starved cells treated with CPT (12.5 μM) for 1 h. **c** 53BP1 foci in serum-starved cells after 1 h treatment with 12.5 μM CPT, and during repair in drug-free medium. **d** Analysis of TOP1 cleavage-complexes (TOP1cc) by ICE assay. Serum-starved cells were treated with CPT (25 μM) for 1 h followed by 1 h repair in drug-free medium. *Top*, quantification. *Bottom*, representative plot of TOP1cc is shown. UNT untreated. *n* = 3 independent experiments. Data were represented as mean ± SEM. Statistical significance was determined by two-tailed unpaired *t*-test for (**b**) and (**d**), and two-way ANOVA followed by Tukey’s multiple comparisons test for (**c**).

Some studies have shown that tyrosyl-DNA phosphodiesterase 2 (TDP2), a TOP2cc debulking enzyme involved in TOP2-induced DSB repair [37–39], can process TOP1-DNA adducts in vitro. Additionally, TDP2 deficiency has been shown to increase CPT hypersensitivity in TDP1-deficient avian, murine and human cells [40, 41]. Recently, Geraud and colleagues demonstrated that TDP2 can back up TOP1-induced DSB repair in G1 U2OS cells [24]. To investigate the participation of TDP2 in TOP1-induced DSB repair in quiescent RPE-1 cells, we depleted TDP2 in both *TDP1*^*+/+*^ and *TDP1*^*-/-*^ cells (Fig. 4a). As previously reported, TOP1 levels remained unchanged, undergoing uniform degradation upon CPT treatment (Supplementary Fig. 5a). While TDP2 depletion did not significantly promote the accumulation of TOP1-induced DSBs in *TDP1*^*+/+*^ cells, it led to a mild increase compared to TDP1-deficient cells (Fig. 4b). Consistently, TDP2 depletion only caused a minor defect (3 h) in TOP1-induced DSB repair, suggesting that TDP2 is dispensable for repairing these breaks in wild-type cells (Fig. 4c). However, strikingly, TDP2 depletion in *TDP1*^*-/-*^ cells exacerbated the DSB repair defect observed in the latter, supporting the idea that TDP2 plays a key role in facilitating the residual DSB repair detected in *TDP1*^*-/-*^ cells (Fig. 4c). This repair defect was suppressed by wild-type TDP1 overexpression (Supplementary Fig. 5b).

Next, we asked whether TDP2 could hydrolyze TOP1ccs during TOP1-induced DSB repair, similar to TDP1. To ensure an appropriate time window, we measured TOP1ccs one hour after CPT removal, when the *TDP1*^-/-^ debulking defect in *TDP1*^*-/-*^ cells is barely detectable (Fig. 2i). Notably, TDP2 depletion in *TDP1*^-/-^ cells further highlighted this defect (Fig. 4d). This repair defect was suppressed by TDP1 overexpression (Supplementary Fig. 5c). Altogether, these results indicate that TDP2 is not required to release TOP1ccs from DNA ends in quiescent RPE-1 cells unless TDP1 is absent.

### TDP1^H263A^ mutant remains associated with abortive TOP1cc

Since the H263A mutation does not trap TDP1 on DNA and its repair kinetics differ from those of H493R, we reasoned that the mechanism of TOP1-induced DSB blockage might be different from that of H493R but somehow linked to the recruitment of TDP1^H263A^ to abortive TOP1ccs. To explore this possibility we analyzed the chromatin recruitment of wild-type TDP1, TDP1^H263A^ and TDP1^H493R^ upon CPT-induced DSB formation and repair (Supplementary Fig. 6a). However, we did not observe significant changes in chromatin recruitment for any TDP1 forms, either upon induction or after twenty-four hours of repair (Supplementary Fig. 6b). Notably, TDP1^H263A^ presented two different electrophoretic mobilities (Supplementary Fig. 6b, c). Strikingly, we observed a very strong increase in the low-mobility form of TDP1^H263A^ on chromatin upon CPT treatment (Supplementary Fig. 6c).

To finely explore whether the H263A mutation might provoke a prolonged non-covalent association of TDP1H263A to abortive TOP1ccs, we analyzed the co-localization of TDP1^H263A^ and TOP1cc by proximity ligation assays (PLA). Upon CPT treatment, both TDP1^H263A^ and TDP1^H493R^ exhibited a slightly increased colocalization with colocalization with TOP1ccs compared to wild-type TDP1 (Supplementary Fig. 6d). Notably, while most of PLA signal decayed during repair, TDP1^H263A^ retained higher colocalization with TOP1ccs than wild-type TDP1 and TDP1^H493R^, whose levels returned to those observed under untreated conditions (Supplementary Fig. 6d). Together with SSB and DSB repair kinetics previously shown in TDP1^H263A^ (Fig. 1c–e & Supplementary Fig. 3), these results suggest that TDP1^H263A^ may persist associated to TOP1-induced DSBs.

### TDP1^H493R^ and TDP1^H263A^ promote genome instability and cell death

**Fig. 5:**
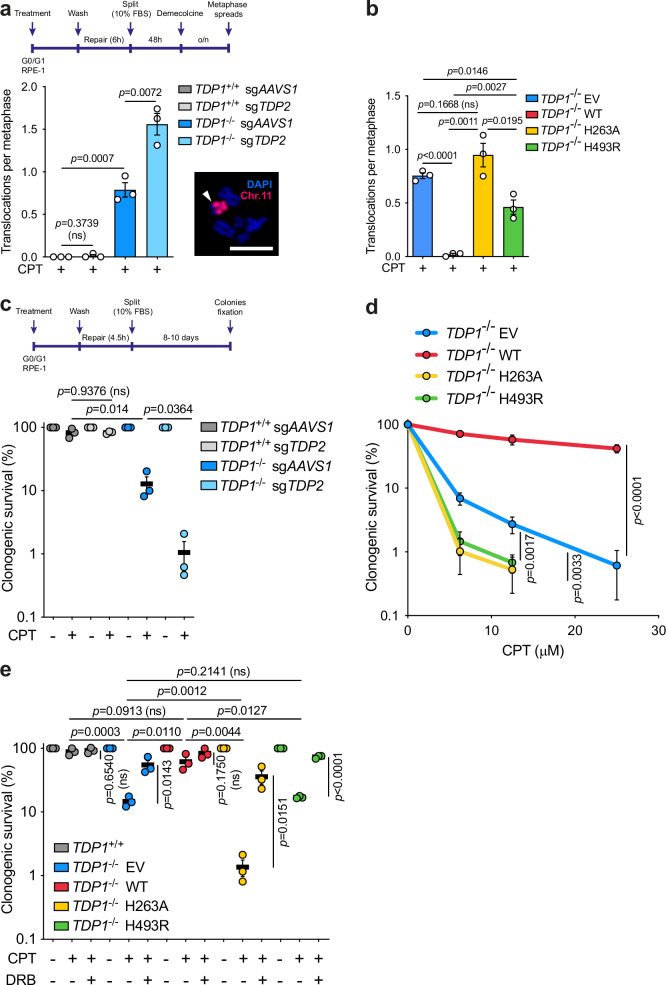
TDP1^H493R^ and TDP1^H263A^ mutations promote genome instability and cell death upon TOP1 poisoning. **a** Translocation frequencies were quantified in serum-starved *TDP1*^+/+^ and *TDP1*^−/−^ mock-depleted (sg*AAVS1*) or TDP2-depleted (sg*TDP2*) RPE-1 cells in metaphase spreads prepared 48 h after CPT treatment (25 μM) for 2 h followed by 6 h repair in drug-free medium. Workflow and a representative image of a chromosomal translocation are shown. White arrow indicates a translocation event. **b** Translocation frequencies were quantified in serum-starved *TDP1*^*-/-*^ RPE-1 cells complemented with an empty vector (EV), wild-type TDP1 (WT), TDP1^H263A^ (H263A) or TDP1^H493R^ (H493R). Other details as in (**a**). **c**–**e** Clonogenic survival of serum-starved *TDP1*^+/+^ and *TDP1*^−/−^ mock-depleted (sg*AAVS1*) or TDP2-depleted (sg*TDP2*) cells (**c**), or *TDP1*^+/+^ and *TDP1*^−/−^ RPE-1 cells complemented with an empty vector (EV), wild-type TDP1 (WT), TDP1^H263A^ (H263A) or TDP1^H493R^ (H493R) (**d**, **e**), treated with CPT (6.25 μM for **c** and **e**) for 2 h, and after 6 h repair in drug-free media. A low doxycycline dose was used in **e**. Where indicated, cells were pre-incubated with DRB (100 μM) for 3 h prior to CPT treatment. After repair, cells were collected and re-cultured in serum-containing media. Workflow is shown in **c**. *n* = 3 independent experiments. Data were represented as mean ± SEM. Statistical significance was determined by two-tailed unpaired *t*-test for (**a–c** and **e**) and by two-way ANOVA followed by Tukey’s multiple comparisons test for (**d**). ns non-significance. Scale bar, 10 μm.

We recently described that TDP1 suppresses chromosomal translocations and cell death induced by abortive TOP1 activity during gene transcription [23]. For a better understanding of the relevance of the removal of the TOP1cc adduct we analyzed potential TOP1-induced DSB repair defects in other SSB repair factors downstream of TDP1. We focused on PNKP, the following enzymatic activity working on TOP1cc repair. We achieved more than 90% depletion of PNKP with CRISPR-Cas9 by a single guide RNA (sgRNA) in two independent clones (Supplementary Fig. 7a). PNKP depletion resulted in a small but significant defect in TOP1-induced DSB repair, similar to that observed in *TDP1*^*−/−*^ cells. These results suggest that restoration of DNA end polarity is relevant in TOP1-induced DSB repair. Next, we studied whether PNKP depletion influenced the formation of transcription-associated chromosomal reorganizations in *TDP1*^*-/-*^ complemented cells upon CPT treatment. Cells were treated and maintained in G0/G1 during six hours after treatment with CPT and then released for the isolation of metaphases [23] (Fig. 5a). However, PNKP-depleted cells did not provoke a significant increase in chromosomal translocations (Supplementary Fig. 7b). These results indicate that while PNKP participates in TOP1-induced DSB repair, it is not essential, being dispensable for the suppression of genome instability. This also suggest that removal of TOP1cc from 3’-DNA termini must be a key step to prevent genome instability induced by TOP1-induced DSBs.

Given that TDP2 backs up TDP1 loss in TOP1-induced DSB repair, we hypothesized that TDP2 may be relevant to suppress genome instability in quiescent TDP1-lacking cells. To test this, we examined the influence of TDP2 in the formation of TOP1-induced chromosomal translocations. Notably, TDP2 depletion in *TDP1*^*-/-*^ cells further increased CPT-induced translocations compared to TDP1-deficient cells alone, whereas no effect was observed in *TDP1*^*+/+*^ cells (Fig. 5a). Altogether, these results suggest that TOP1cc removal is a key step to prevent genome instability generated by TOP1-induced DSBs in quiescent cells.

Next, to estimate the physiological relevance of TDP1^H263A^ and TDP1^H493R^ blockage of TOP1-induced DSB repair, we studied the formation of chromosomal reorganizations in *TDP1*^*-/-*^ complemented cells. While wild-type TDP1 suppressed chromosomal translocations induced by CPT, TDP1^H263A^ did not (Fig. 5b). TDP1^H493R^ showed an intermediate phenotype (Fig. 5b). Notably, TDP1^H263A^-expressing cells showed a higher accumulation of chromosomal translocations than both EV and TDP1^H493R^-expressing cells, being the latter the one that accumulated the least (Fig. 5b). These translocations correlated with relative accumulation of TOP1-induced DSB upon CPT treatment (Fig. 1b), reinforcing the link between TOP1-induced DSBs and chromosomal translocations.

Finally, to study the contribution of TDP1^H263A^ and TDP1^H493R^ to CPT-induced cytotoxicity in quiescent cells, we performed clonogenic survival in RPE-1 cells that had been treated with CPT, allowed to repair while quiescent and finally transferred to serum-containing medium (Fig. 5c). As we had previously shown, *TDP1*^*−/−*^ cells exhibited a high sensitivity to CPT (Fig. 5c and Supplementary S5d). This hypersensitivity was significantly exacerbated by TDP2 depletion, in agreement to our results on DSB repair kinetics and chromosomal translocations, and further confirming that TDP2 backs up TDP1 in RPE-1 quiescent cells (Fig. 5c and Supplementary S5d). Next, we analyzed clonogenic survival in TDP1^H263A^ and TDP1^H493R^-complemented *TDP1*^*−/−*^ cells. Strikingly, both mutants failed to rescue CPT sensitivity (Fig. 5d). This effect was limited to TDP1-deficient cells, since TDP1^H263A^ and TDP1^H493R^ expression in *TDP1*^*+/+*^ cells did not promote any significant defect (Supplementary Fig. 8). We obtained similar results with reduced doxycycline concentrations, ruling out potential effects due to overexpression of TDP1 mutants (Fig. 5e). Importantly, RNA polymerase II elongation inhibition with DRB prior to TOP1 poisoning suppressed CPT sensitivity in EV, TDP1^H263A^ and TDP1^H493R^-expressing cells (Fig. 5e). Altogether, these results demonstrate that TDP1^H263A^ and TDP1^H493R^ promote toxicity by TOP1-induced DSBs associated with gene transcription in quiescent cells. Furthermore, the H263A mutation appears to be more deleterious than both TDP1 loss and the H493R mutation.

## Discussion

Postmitotic cells are highly susceptible to DNA damage, and defects in several SSB repair factors result in hereditary neurological syndromes [6]. A common source of SSBs is the abortive activity of TOP1 during gene expression. Notably, transcription-associated TOP1-induced SSBs can also be converted into DSBs, a much more cytotoxic DNA lesion [17]. Nevertheless, the relevance of transcription-associated TOP1-induced DSBs in the pathology of these diseases remains unclear. In this study, we addressed the impact of the H263A and the H493R (SCAN1) mutations in TDP1 on the repair of TOP1-induced DSBs in quiescent cells. We previously reported that the loss of TDP1 significantly delays TOP1-induced DSB repair in quiescent RPE-1 cells, promoting genome instability and cell death [23]. Remarkably, our complementation experiments on *TDP1*^*-/-*^ RPE-1 cells showed that TDP1^H493R^ expression not only fails to suppress this delay but also results in fully defective TOP1-induced DSB repair in quiescent cells, in agreement with a similar observation in G1 U2OS cells [24]. To our understanding, this is a very significant discovery that demonstrates the robustness of these new findings and highlights an important particularity of the SCAN1-causing mutation compared to TDP1 loss.

**Fig. 6:**
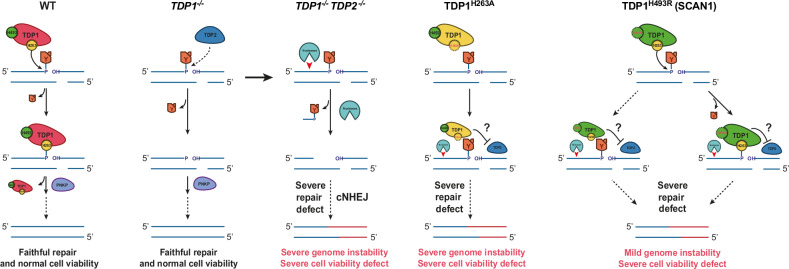
Model for TOP1-induced DSB repair and TDP1^H493R^ and TDP1^H263A^-associated genome instability and reduced survival.

Importantly, *TDP1*^-/-^ and TDP1^H493R^-expressing RPE-1 quiescent cells accumulated similar levels of TOP1ccs upon CPT treatment. Additionally, in agreement with previous reports [5, 25, 35], we observed that H493R mutation impairs the resolution of the TDP1-DNA phosphohistidyl intermediate by trapping TDP1^H493R^ covalently to the DNA in vivo. Since *TDP1*^-/-^ and TDP1^H493R^ cells showed similar levels of abortive TOP1ccs, we suggest that trapped TDP1^H493R^-DNA complexes must be much less frequent than abortive TOP1ccs. This agrees with the low efficiency of TDP1^H493R^ at the first step of TOP1ccs removal [11, 12]. Nevertheless, despite this low frequency, significant levels of trapped TDP1^H493R^ remained after repair, even at twenty-four hours. This result is surprising considering the extremely low half-life of the TDP1^H493R^-DNA (single-stranded) complex in vitro [14, 42]. However, it agrees with a previous report demonstrating TDP1^H493R^ trapping in mitochondrial DNA [35]. Importantly, together with the rapid decrease of PAR observed in TDP1^H493R^-expressing cells, these results suggest that trapped TDP1^H493R^ is likely associated with DSBs and conduct us to propose that, additionally to remaining abortive TOP1ccs, TDP1^H493R^ trapping contributes to TOP1-induced DSB repair blockage in TDP1^H493R^-expressing cells (Fig. 6).

Previous studies have shown that TDP2 can process TOP1-DNA adducts in vitro, and that TDP2 deficiency increases hypersensitivity to CPT in TDP1-deficient avian, murine and human cells [40, 41, 43]. Importantly, TDP2 can back up TOP1-induced DSB repair in G1 U2OS cells [24]. However, the possible role of TDP2 on TOP1-induced DSB repair in quiescent cells had not been studied. Notably, depletion of TDP2 in *TDP1*^*-/-*^ cells resulted in a very significant reduction of repair rates, suggesting that TDP2 can back up TDP1 in TOP1-induced DSB repair in quiescent RPE-1 cells (Fig. 6). Otherwise, we did not detect repair deficiencies in the long term when TDP2 was depleted in a TDP1-proficient background. This is consistent with the weak 3’-tyrosyl-DNA phosphodiesterase activity of TDP2 compared to TDP1 and with TDP1 sufficiency for TOP1-induced SSB repair and CPT resistance in human, murine and avian cells [40, 41, 43]. These results would suggest that TDP2 is physiologically irrelevant in the repair of TOP1-induced DSBs when TDP1 is available (Fig. 6). On the other hand, the fact that trapped TDP1^H493R^ is efficiently released when expressed in TDP1-proficient cells but not in *TDP1*^*-/-*^ cells, despite the availability of TDP2 in both, implies that only TDP1 is able to suppress SCAN1-associated TOP1-induced DSB repair blockage, in agreement with the recessive nature of SCAN1. These results agree with the observation that, in G1 U2OS cells, TDP1 depletion in SCAN1 cells restores the ability of cells to reverse 53BP1 foci upon CPT removal, while concurrent depletion of TDP1 and TDP2 leads back to defective repair [24]. These results could indicate that, contrary to wild-type TDP1, TDP2 is unable to unlink trapped TDP1^H493R^ in vivo, and/or that TDP2 is not sufficiently active on 3’-TOP1 termini to out-compete or prevent their non-productive binding by TDP1^H493R^ (Fig. 6).

In this work, we also complemented *TDP1*^*-/-*^ cells with TDP1^H263A^, a different catalytically inactive mutant of TDP1 [5, 25]. We failed to obtain high levels of TDP1^H263A^ expression in *TDP1*^*-/-*^ and *TDP1*^*+/+*^ cells, in agreement with previous reports showing an inherent toxicity of TDP1^H263A^ expression in yeast and human cells [27–29]. By 53BP1 repair kinetics, we detected a minor defect in TOP1-induced DSB repair after twenty-four hours. To our surprise, direct study of DSB by neutral comets revealed a much more significant defect than in 53BP1 repair kinetics, which was confirmed by direct measurement of chromosomal breaks in quiescent cells. Considering that neutral comets are a direct measurement of DSBs, we speculate that the weaker defect in the disappearance of 53BP1 foci in TDP1^H263A^, compared to TDP1^H493R^-expressing cells, might reflect some defect in DSB signaling. However, the clarification of this phenotype requires further investigation.

Notably, we detected a significant accumulation of abortive TOP1cc but not TDP1 trapping in TDP1^H263A^ cells. One possibility is that the repair defect in TDP1^H263A^ is induced by a different mechanism than in TDP1^H493R^. Another possibility is that it is non-covalently trapped TDP1 on TOP1ccs that causes the DSB repair defect in both cases. This would explain the big repair defect detected by comets in TDP1^H263A^ compared to TDP1^H493R^, since it is less able to hydrolyze TOP1-DNA linkages than TDP1^H493R^ [25]. Indeed, overexpression of TDP1^H263A^ in a wild-type background significantly increased the accumulation of CPT-induced DSBs, although it did not result in significant TOP1cc accumulation. An interesting observation is that while TDP1^H263A^ does not get covalently trapped to DNA, it remains associated to TOP1cc long after CPT wash, suggesting a possible mechanism of interference with TDP1-alternative hydrolytic factors (Fig. 6). Intriguingly, in our chromatin recruitment experiments, we noticed the appearance of two TDP1^H263A^ bands with very similar electrophoretic mobility, that could be explained by the fact that TDP1 suffers a variety of post-translational modifications [44]. Indeed, TDP1-S81 phosphorylation promotes DNA repair in response to CPT-induced DSBs, being required for its focal accumulation [45]. However, the characterization of the effect of this mutation requires further investigation.

We recently showed that TDP1-dependent DSB repair suppresses CPT-induced chromosome translocations generated by TOP1 abortive activity, demonstrating that TOP1-induced DSBs during transcription can result in replication-independent genome reorganizations [23]. Depletion of PNKP, despite causing a delay in repair similar to that of TDP1 loss, did not promote any increase in these reorganizations. These results are similar to those obtained when PARP1 is inhibited [23] and strongly suggest that the debulking of abortive TOP1cc is the key step to prevent unfaithful repair and genome reorganizations. Notably, complementation with wild-type TDP1 but not with TDP1^H263A^ or TDP1^H493R^ suppressed CPT-induced chromosomal translocations in *TDP1*^*-/-*^ cells, indicating that either more persistent abortive TOP1cc or covalent DNA-TDP1 complexes promote these translocations and suggesting that abortive TOP1cc could be a source of genome instability in SCAN1 cells (Fig. 6). Translocation levels were significantly lower in TDP1^H493R^-expressing cells compared to EV and TDP1^H263A^, potentially reflecting the reduced accumulation of DSBs that we attribute to the residual debulking activity of TDP1^H493R^. We also tested the formation of these reorganizations depleting TDP2 in wild-type and TDP1-lacking cells. Notably, TDP2 depletion further increased genome reorganizations in TDP1-lacking cells, indicating that TDP2-dependent repair of TOP1-induced DSBs is also protective against unfaithful repair of abortive TOP1ccs (Fig. 6). This is not surprising since TDP2 is expected to enzymatically replace TDP1 activity on abortive TOP1cc, producing the same clean DNA ends [43]. Importantly, the repair defect caused by TDP2 depletion in *TDP1*^-/-^ cells was not complete, suggesting that alternative but likely less efficient pathways can process TOP1 in the absence of TDP1 and, more often, in the absence of both TDP1 and TDP2 (Fig. 6). Several nucleases, such as MRE11, CtIP, XPF, APE2, and MUS81, have been shown to mediate resection in abortive TOP1cc intermediates [4]. We propose that the removal of abortive TOP1ccs by TDP1 or TDP2 would initiate a conservative TOP1-induced DSB repair pathway, while nucleases, such as MRE11 and subsequent canonical non-homologous end-joining (cNHEJ) [23] would promote genome instability and cell death (Fig. 6). The blockage of the TDP2-dependent pathway by TDP1^H263A^ and TDP1^H493R^ would also promote these error-prone pathways (Fig. 6).

A remarkable result of this study is the contribution of TDP1^H493R^ expression in the cytotoxicity provoked by replication-independent abortive TOP1 activity. We took advantage of our cellular system to analyze the toxicity of abortive TOP1 cycles in quiescent cells. Quiescent *TDP1*^*−/−*^ RPE-1 cells are very sensitive to transcription-associated abortive TOP1 activity [23]. Importantly, here we demonstrate that TDP1^H493R^ or TDP1^H263A^ expression results in a further decrease in survival (Fig. 6). Additionally, our results suggest that, somehow, abortive TOP1-induced DSBs would be more resistant to repair in SCAN1 cells than TOP1-induced SSBs. This is a relevant observation since SSB repair defects are a source of cell death in the brain, and TDP1 facilitates chromosomal SSB repair in neurons and is neuroprotective in vivo [16]. Importantly, defects in TDP1, PARP1, XRCC1, PNKP, and LIG3 increase the formation of replication-independent CPT-induced DSBs [21, 23], suggesting that the formation of TOP1-induced DSBs is dependent on the accumulation of unrepaired TOP1-induced SSBs. Our results support the idea that transcription-associated TOP1-induced DSBs must be a relevant part of the pathological events underlying SCAN1 and other related diseases.

Altogether, we propose a model in which TDP1 is the major TOP1cc debulking factor of transcription-associated TOP1-induced DSBs in RPE-1 quiescent cells. TDP2, can backup TDP1, but not completely. Thus, TDP1-deficient cells are defective in TOP1-induced DSB repair, accumulate genome instability and have decreased survival. However, TDP2 loss in TDP1-deficient cells is especially deleterious, suggesting that alternative debulking pathways are insufficient and error-prone. H263A mutant is persistent at TOP1cc associated with DSBs but unable to efficiently remove these adducts. Somehow, it blocks alternative debulking pathways, blocking DSB repair and promoting genome instability and reducing cellular survival in quiescent cells. Finally, SCAN1 mutation H493R does not accumulate significant TOP1cc but TDP1-DNA covalent complexes associated with DSBs, and blocks alternative debulking pathways. Wild-type TDP1 can outcompete mutant TDP1, rendering these mutations recessive. In contrast, TDP2 cannot compensate, though the reason for this remains unclear, leading to genome instability and compromised cell survival in homozygous H263A and H493R mutants due to TOP1 abortive activity during transcription (Fig. 6).

In summary, our results uncover a novel deleterious effect of H263A and H493R TDP1 mutants in response to TOP1 abortive cycles in quiescent cells, highlighting the threat posed by TOP1-induced DSBs during transcription to SCAN1 cells with important implications in SSB-repair-associated neuropathology.

## Methods

### Cell lines and culture conditions

hTERT RPE-1 cells (originally purchased from ATCC, CRL-4000) were propagated in DMEM/F12 medium supplemented with 10% fetal bovine serum (FBS) and with 1% penicillin and streptomycin. For serum starvation, cells were grown until confluency, washed twice with serum-free media, and then cultured in 0% FBS for 3–6 days.

TDP1 complementation in *TDP1*^*-/-*^ and *TDP1*^*+/+*^ RPE-1 cells was achieved by lentiviral infection. Lentiviral particles were obtained from piFLAG-NEO, piFLAG-TDP1, piFLAG-TDP1:H263A and piFLAG-TDP1:H493R vectors (this work). Infected cells were selected in G418 for 10 days. For inducing protein expression, doxycycline (SIGMA) was added at 0.1 µg/ml 24 h before experiments unless indicated. Low expression of TDP1 variants was achieved with 0.01 µg/ml of doxycycline for wild-type TDP1 and TDP1:H263A and with 0.005 µg/ml of doxycycline for TDP1:H493R (indicated as *low doxycycline dose* in figure legends). Pooled cells were tested for the expression of TDP1 variants by western blotting.

For sgRNA-mediated stable depletion of PNKP or TDP2, cells were infected with lentiviral particles generated using the vector #52961 (AddGene) and selected with 20ug/ml puromycin for 24–48 h. Pooled cells were tested for the loss of PNKP or TDP2 expression by western blotting. Target sequences used in guide RNAs are: CGTATGCGGAAGTCAAACCC for *PNKP* and TCTGTCAGAGAGGGCTCGAG for *TDP2*.

All cell lines were grown at 37 °C, 5% CO_2_ and were regularly tested for mycoplasma contamination. All cell lines tested negative for mycoplasma contamination.

### Western blotting

Protein extracts were obtained by lysing cell pellets at 100 °C for 10 min in 2x protein buffer (125 mM Tris, pH 6.8, 4% SDS, 0.02% bromophenol blue, 20% glycerol, 200 mM DTT). Extracts were then sonicated in a Bioruptor (Diagenode) for 1 min at high intensity. Primary antibodies were blocked in Tris buffered saline buffer, 0.1% Tween20, 5% BSA and employed as follows: TDP1 (Santa Cruz B, sc-365674) 1:250, Vinculin (Santa Cruz B, sc-25336) 1:1000, TDP2 [39] 1:5000, PNKP (AbCam, ab170954) 1:1000, FLAG-M2 (Merk, F1804), H3 (AbCam, ab1791), Tubulin (AbCam, ab15568). Vinculin was used as a loading control. Secondary antibodies (1:5000 dilution in Tris buffered saline buffer 0.1% Tween20 5% BSA): HRP-bovine anti-goat IgG (H + L), HRP-goat anti-mouse IgG (H + L) and HRP-goat anti-rabbit IgG (H + L) (Jackson ImmunoResearch 805-035-180, 115-035-146 and 115-035-144, respectively).

Chemiluminescence data were collected on a ChemiDoc imaging system and analyzed in Image Lab 6.0.0 (BIO-RAD) and Image J. Molecular weight reference is in KDa.

### Immunofluorescence and FISH

For immunofluorescence (IF), cells were grown on coverslips for 4-7 days and then treated as indicated. Cells were fixed (10 min in PBS–4% paraformaldehyde), permeabilized (5 min in PBS–0.2% Triton X-100), blocked (30 min in PBS–5% BSA), and incubated with the indicated primary antibodies for 1–3 h or o/n in PBS–1% BSA. Cells were then washed (3 × 5 min in PBS– 0.1% Tween20), incubated for 30 min with the corresponding AlexaFluor-conjugated secondary antibody (1:1000 dilution in PBS–1% BSA) and washed again as described above. Finally, cells were counterstained with DAPI (Sigma, D9542) and mounted in antifade mounting medium for fluorescence (Vectashield, Vector Labs, H-1000). Primary antibodies: 53BP1 (Novus Biologicals, NB100-904) 1:2500, PAR (Millipore, MABE1016) 1:1000. Secondary antibodies: Alexa Fluor 488-goat anti-mouse IgG (H + L), Alexa Fluor 488-goat anti-rabbit IgG (H + L), Alexa Fluor 546-goat anti-mouse IgG (H + L), Alexa Fluor 546-goat anti-rabbit IgG (H + L) (ThermoFisher Scientific A11001, A11008, A11003 and A11010, respectively). Whole chromosome FISH was performed according to manufacturer’s protocol (MetaSystems probes, Whole Chromosome Paint, 739D-0308-050-FI & 739D-0311-050-OR).

Fluorescence intensity of nuclear PAR was obtained using ImageJ 1.52 d. DAPI signal was used to delimit the nucleus, and the intercellular background was subtracted.

### 53BP1 repair kinetics

53BP1 foci were scored manually (double blind) in untreated conditions, after treatment with drugs, and during repair in drug-free medium. 53BP1 foci were manually counted (double-blind) in 20–40 cells per data point per independent experiment. Values are shown as the average of 53BP1 foci per cell relative to treatment.

### Metaphase spreads

For metaphase spreads, cells were incubated with demecolcine (Sigma) at 0.2 mg/ml for 6–20 h and then harvested. Cells were collected using standard cytogenetic techniques, subject to hypotonic shock for 1 h at 37 °C in 0.03 M sodium citrate and fixed in 3:1 methanol:acetic acid solution. Fixed cells were dropped onto acetic acid-humidified slides before dehydration and FISH.

### Chromosomal translocations

Translocation frequencies were calculated as translocations per metaphase in chromosomes 8 and 11, scored manually (double-blind) and plotted together.

### Premature chromosome condensation (PCC)

For premature chromosome condensation (PCC), G0/G1 RPE-1 cells were fused to HeLa cells synchronized in metaphase. To do this, demecolcine was added to cycling HeLa cells. Mitotic HeLa cells were collected by mitotic shake-off. Independently, serum-starved RPE-1 cells were treated as indicated and collected by trypsinization. HeLa and RPE-1 cells were mixed in a 1:5 ratio. Cell mixture was then centrifuged and resuspended in 50% (w/v) polyethylene glycol (PEG) 1500 (Sigma) prepared in serum-free media. Cell mixture was centrifuged and the pellet was resuspended in serum-free media with demecolcine and incubated at 37 °C for 1 h. After that, the cell mixture was washed with PBS, and hypotonic shock and cell fixation were performed as described for metaphase spreads. Chromosomal breaks were scored in Giemsa-stained metaphase spreads.

### Clonogenic survival assays

For quiescent cells, G0/G1 cells were treated as indicated, then washed, trypsinized and counted. A total of 400 cells were re-cultured in serum-containing media and grown for 8–10 days. In all cases, cells were fixed and stained in PBS-70% ethanol/1% methylene blue. Colonies were counted manually (double blind). The surviving fraction at each dose was calculated by dividing the average number of colonies in treated dishes by the average number in untreated dishes. In all biological replicates, cells were split in duplicate for each experimental condition.

### Cell cycle analysis

Cells were incubated with 10 µM BrdU (Sigma, B5002) for 15 min. Cells were washed twice with PBS and fixed with 70% ethanol overnight. DNA was denatured with 2 N HCl/Triton X-100. Cells were incubated with anti-BrdU (Santa Cruz, sc-32323) at 1:1000 overnight at 4 °C. After that, AlexaFluor-conjugated secondary antibody (Invitrogen) was added at 1:1000 during 1 h. Finally, before flow cytometry, cells were incubated with 100 mg/ml PI and 100 mg/ml RNAse A for 30 min. Data were collected in a BD FACSCanto II flow cytometer and analyzed in BD FACSDiva Software v9.0.

### ICE assay

ICE assay was performed as previously described [34] with minor modifications. Briefly, a total of 2 × 10^6^ cells were treated as indicated and lysed with 3 ml 1% Sarkosyl. The lysate was passed through a 25G5/8 gauge ten times. 2 ml of CsCl solution (1.5 g/ml) was layered into an ultracentrifugation tube. The volume of lysate was layered on top of the gradient. Samples were centrifuged in a NVt90 rotor at 25 °C, 121,900 g for 20 h. Pellet was resuspended in TE 1× and DNA concentration was measured in a Nanodrop. 1–10 µg of DNA was loaded in nitrocellulose membrane preincubated in 25 mM NaPO_4_ pH 6.5 for 15 min using a slot-blot apparatus. Abortive TOP1cc were detected by TOP1cc antibody [36] (Millipore, MABE1084) 1:250 and DNA-TDP1^H493R^ covalent complexes by FLAG antibody (Merck, F1804).

### Comet assay

Cells were grown until confluency, then serum-starved for three days, collected and treated in suspension in 0% FBS medium as indicated. After treatment, cells were washed once and resuspended in 0.5 ml ice-cold PBS. Neutral comet assay was performed as previously described (23, 40). Briefly, cells were mixed with an equal volume of 1.2% low-melting-point agarose (Lonza, 50080) in PBS (at 42 °C). Cell suspension was immediately layered onto pre-chilled frosted glass slides pre-coated with 0.6% agarose and maintained in the dark at 4 °C until agarose set. Slides were then immersed in pre-chilled lysis buffer (2.5 M NaCl, 10 mM Tris–HCl, 100 mM EDTA, 1% N-laurosylsarcosine, 10% v/v DMSO, 0.5% v/v Triton X-100, pH 9.5) at 4 °C for 1 h. Cells were washed three times and incubated with pre-chilled electrophoresis buffer (300 mM sodium acetate, 100 mM Tris-HCl, bring up to pH 8.3) for 1 h at 4 °C. Electrophoresis was run at 0.5 V/cm for 1 h. Following electrophoresis, slides were incubated in 0.4 M Tris-HCl pH 7 for 1 h before SYBR green staining.

Slides were visualized by using a fluorescence microscope (Olympus BX-61). Values are shown as the quantification of comet tail moments. In all cases, experiments were analyzed by CometScore Pro software.

### Biochemical fractionation

Fractionation experiments were carried out as previously described [46]. In brief, cells were washed and collected in pre-chilled PBS by scraping. One-tenth of the total volume was saved as total extract. The rest was spun at hight speed and resuspended in pre-chilled lysis buffer (50 mM HEPES pH7.5, 150 mM NaCl, 1 mM EDTA, 0.1% triton), including protease inhibitors (Merck, 11697498001) and phosphatase inhibitors (Merck, P0044) for 5 min on ice. Insoluble material was pelleted at maximum speed for 5 min, and the supernatant was saved as the soluble fraction. Pellet was resuspended and washed twice in lysis buffer and resuspended as the chromatin fraction. All fractions were boiled in 2X protein buffer and sonicated as indicated above.

### Proximity ligation assay (PLA)

Duolink PLA assay (Sigma, DUO92101) was performed according to the manufacturer’s instructions. Briefly, cells were grown on coverslips until confluency, then serum-starved for 3 days and treated as indicated. Cells were fixed (10 min in PBS–4% paraformaldehyde), permeabilized (5 min in PBS–0.2% Triton X-100), blocked (30 min in Duolink blocking solution), and incubated with the required primary antibodies for 90 min in Duolink antibody diluent at 37 °C. After that, incubation with secondary antibodies conjugated with oligonucleotides was performed, followed by ligation and amplification. Finally, coverslips were mounted with Duolink in situ mounting medium with DAPI. Primary antibodies were used as indicated for IF, ICE, and western blotting. TDP1 (Proteintech, 10641-1-AP) and TOP1cc (Millipore, MABE1084) antibodies were used.

### Statistical analysis

Statistical analysis is included in figure legends. In all cases, comparison tests were performed using GraphPad Prism version 10.4.0 for macOS. We chose three biological replicates based on the fact that this sample size provides enough power to detect expected effects and in agreement with the common practice of the field, balancing statistical robustness with feasibility. To the best of our knowledge, the data meet the assumptions of the test, including the variation within each group.

## Supplementary information

Supplementary Figure 1

Supplementary Figure 2

Supplementary Figure 3

Supplementary Figure 4

Supplementary Figure 5

Supplementary Figure 6

Supplementary Figure 7

Supplementary Figure 8

Original data

Source data

## Data Availability

The data underlying this article will be shared on reasonable request to the corresponding author. Uncropped blots and source data are provided with this paper.
